# NEDD9 stimulated MMP9 secretion is required for invadopodia formation in oral squamous cell carcinoma

**DOI:** 10.18632/oncotarget.25347

**Published:** 2018-05-22

**Authors:** Stéphane Grauzam, Amanda M. Brock, Casey O. Holmes, Jessica A. Tiedeken, Samantha G. Boniface, Bailey N. Pierson, Daniel G. Patterson, Sonya D. Coaxum, David M. Neskey, Steven A. Rosenzweig

**Affiliations:** ^1^ Department of Cell and Molecular Pharmacology and Experimental Therapeutics, Medical University of South Carolina, Charleston, SC 29425, USA; ^2^ Department of Otolaryngology, Head and Neck Surgery, Medical University of South Carolina, Charleston, SC 29425, USA; ^3^ Hollings Cancer Center, Medical University of South Carolina, Charleston, SC 29425, USA

**Keywords:** invasion, metastasis, invadopodia, matrix metalloproteinases, NEDD9

## Abstract

Neural precursor cell expressed developmentally downregulated 9 (NEDD9) is a component of the metastatic signatures of melanoma, breast cancer, glioblastoma, lung cancer and head and neck squamous cell carcinoma (HNSCC). Here we tested the efficacy of NEDD9's domains in stimulating matrix metalloproteinase (MMP) secretion and invadopodia formation in cells stably expressing various NEDD9 mutants. Replacement of the 13 YxxP motif substrate domain (SD) tyrosines and the C-terminal Y629 with phenylalanines (F14NEDD9) eliminated tyrosine phosphorylation, MMP9 secretion and loss of invadopodia formation. Mutation of the N-terminal SH3 domain Y12 to glutamic acid (Y12ENEDD9) or phenylalanine (Y12FNEDD9) reduced MMP9 secretion and inhibited invadopodia formation. SH3 domain deletion (∆SH3NEDD9) resulted in the loss of MMP9 secretion and a lack of invadopodia formation. The SH3–SD domain (SSNEDD9) construct exhibited tyrosine phosphorylation and stimulated MMP9 secretion, as did ∆CTNEDD9 which lacked the C-terminus (∆C-terminal; ∆CT). E13NEDD9 expression blocked MMP9 secretion and invadopodia formation. MICAL1 (molecule interacting with Cas-L1) silencing with a short hairpin RNA reduced MMP9 secretion, vimentin and E-cadherin levels while increasing N-cadherin and Rab6 levels, consistent with reduced invasive behavior. These findings indicate that NEDD9 SD phosphorylation and SH3 domain interactions are necessary for increasing MMP9 secretion and invadopodia formation.

## INTRODUCTION

Cell migration and invasion are highly orchestrated, overlapping processes with invasion representing a degradative form of migration resulting from increased proteinase secretion and proteolysis of extracellular matrix (ECM) proteins within the stroma. Invasion is a property of cancer cells, providing a mechanism by which they exit the primary tumor, traverse the stroma and access the vasculature/lymphatics for distal metastasis. Accordingly, invasive cancer cells must be capable of assembling invadosome structures termed invadopodia that represent sites of focal matrix metalloproteinase (MMP) secretion [1]. Invadopodia are identifiable in distinct two-dimensional *in situ* gelatinase/zymography assays as ventral protrusions of cells with a characteristic central actin fiber core visualized as phalloidin-stained F-actin puncta that overly “black holes” that develop in the fluorescently labeled substratum due to proteolysis [2]. In addition to forming invadopodia and secreting MMPs, invasive cancer cells generate traction force at the rear, with the protrusive process of invadopodia formation occurring at the front, enabling cells to propel through the degraded ECM/stromal barrier. In addition to propulsive force generation, this process requires coordinated substratum attachment and detachment of cells coordinated via focal adhesions [2].

In humans the MMPs represent a large family of at least 24 zinc-dependent endopeptidases that is divided into 4 subgroups based on domain composition [3]. The gelatinase subfamily of MMP2, MMP9 and membrane type 1-MMP, (MT1-MMP or MMP14) are most frequently associated with invadopodia and stroma degradation [4, 5]. In addition to invasion and metastasis, MMP2 and MMP9 have roles in angiogenesis, epithelial to mesenchymal transition (EMT) [6] and histone H3 N-terminal tail cleavage during osteoclastogenesis [7]. Owing to their roles in cell invasion, invadopodia are believed to be the sites of focal secretion of MMP2 and MMP9 along with the localization of MT1-MMP at invadopodia membranes [8].

**Figure 1:**
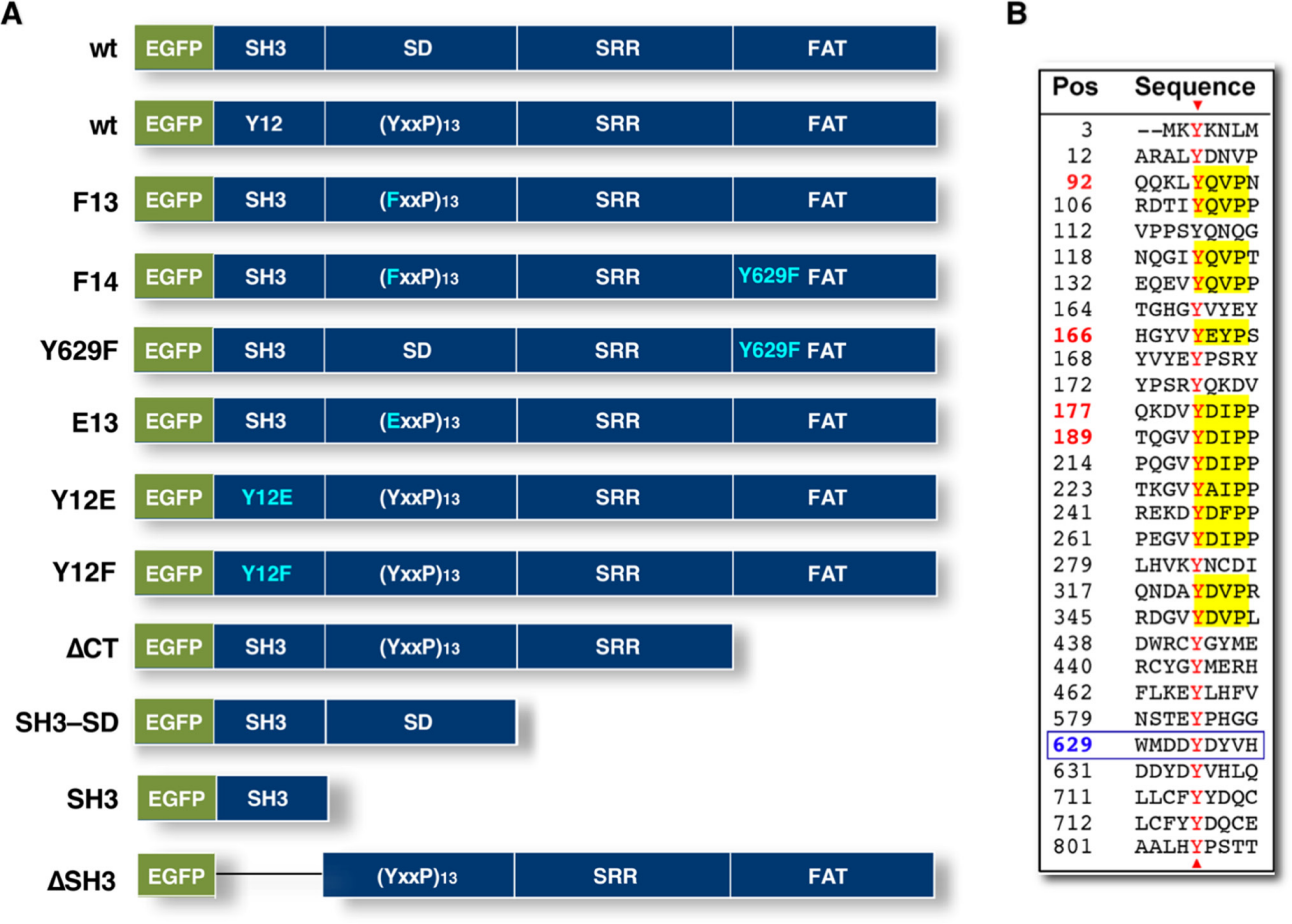
NEDD9 domains and mutant constructs (**A**) NEDD9 structure indicating location of N-terminal EGFP and tyrosines modified in the various constructs described. (**B**) Positions of all 29 NEDD9 tyrosine residues. Those highlighted in yellow were mutated to phenylalanine (F) as indicated for F13 and F14 NEDD9.

Invasion and metastatic disease represent the underlying cause of morbidity and mortality for most solid tumors [9, 10]. However, the molecular details underlying the cellular changes leading to invasion and metastatic disease are incompletely understood and may represent the targets of future therapeutic strategies. We previously demonstrated that neural precursor cell expressed developmentally downregulated 9 (NEDD9; human enhancer of filamentation 1, HEF-1; Crk-associated substrate in Lymphocytes, CasL) is an important regulatory protein involved in head and neck squamous cell carcinoma (HNSCC) cell signaling, leading to migration and invasion [11]. In cells stimulated with VEGF, NEDD9 is rapidly tyrosine phosphorylated within its substrate domain (SD; Figure 1A) in a Src kinase-dependent manner resulting in cell migration, invadopodia formation, MMP9 secretion and invasion; NEDD9 silencing decreased these functions [11]. NEDD9 serves as a scaffold protein within focal adhesions (FAs; [12]) in addition to its obligatory role in matrix metalloproteinase (MMP) secretion, invadopodia formation and cell invasion [11]. Consistent with this role, NEDD9 was identified as a component of the metastatic signatures of HNSCC [13] glioblastoma [14] breast cancer [15] lung cancer [16] and melanoma [17]. In melanoma cells, elevated NEDD9 signaling leads to cell elongation, increased mesenchymal and decreased amoeboid cell migration [18].

While invadopodia and FAs perform different functions, they have a number of proteins in common, including paxillin, cortactin and focal adhesion kinase (FAK) [19]. FAs have also been reported to exhibit degradative activity resulting from recruitment of MT1-MMP via p130Cas/BCAR1 (Breast cancer anti-estrogen resistant) in a complex with FAK [19]. p130Cas is a structurally similar paralog of NEDD9, with both containing N-terminal SH3 domains, followed by a substrate domain (SD), serine-rich region (SRR) and C-terminal focal adhesion targeting (FAT) domain (Figure 1A [20]). Janostiak and coworkers [21] reported that Tyr12 (Y12) in p130Cas is phosphorylated in Src-transformed cells, altering its ability to target FAK to FAs. Cells expressing the phosphomimetic mutant Y12Ep130Cas exhibited decreased, while cells expressing the unphosphorylatable mutant Y12Fp130Cas exhibited increased FA formation and FAK delivery. A shift in presence of tyrosine phosphorylated proteins from FAs to invadopodia was found to be related to decreased FAK in FAs along with elevated function at invadopodia [22, 23]. Unlike NEDD9, p130Cas was not identified as a component of the metastatic signature of melanoma [17]. Collectively, these findings suggest both common and disparate roles for NEDD9 and p130Cas in regulating FAK activity and invadopodia structure and function. On this basis, we wished to further define the requirement of NEDD9, its structural domains and modification by tyrosine phosphorylation in regulating MMP secretion and invadopodia formation. Here we report that MMP9 secretion is associated with invadopodia formation whereas MMP2 secretion is not, suggesting different roles and trafficking patterns for these two MMPs. Moreover, NEDD9 expression, SD tyrosine phosphorylation and an intact SH3 domain are required for invadopodia formation. We further determined that NEDD9 associates with MICAL1 and that MICAL1 silencing leads to cellular changes consistent with mesenchymal to epithelial transition.

## RESULTS

### Mutation of NEDD9 substrate domain (SD) tyrosines to phenylalanine reduces MMP9 secretion and invadopodia formation

**Figure 2:**
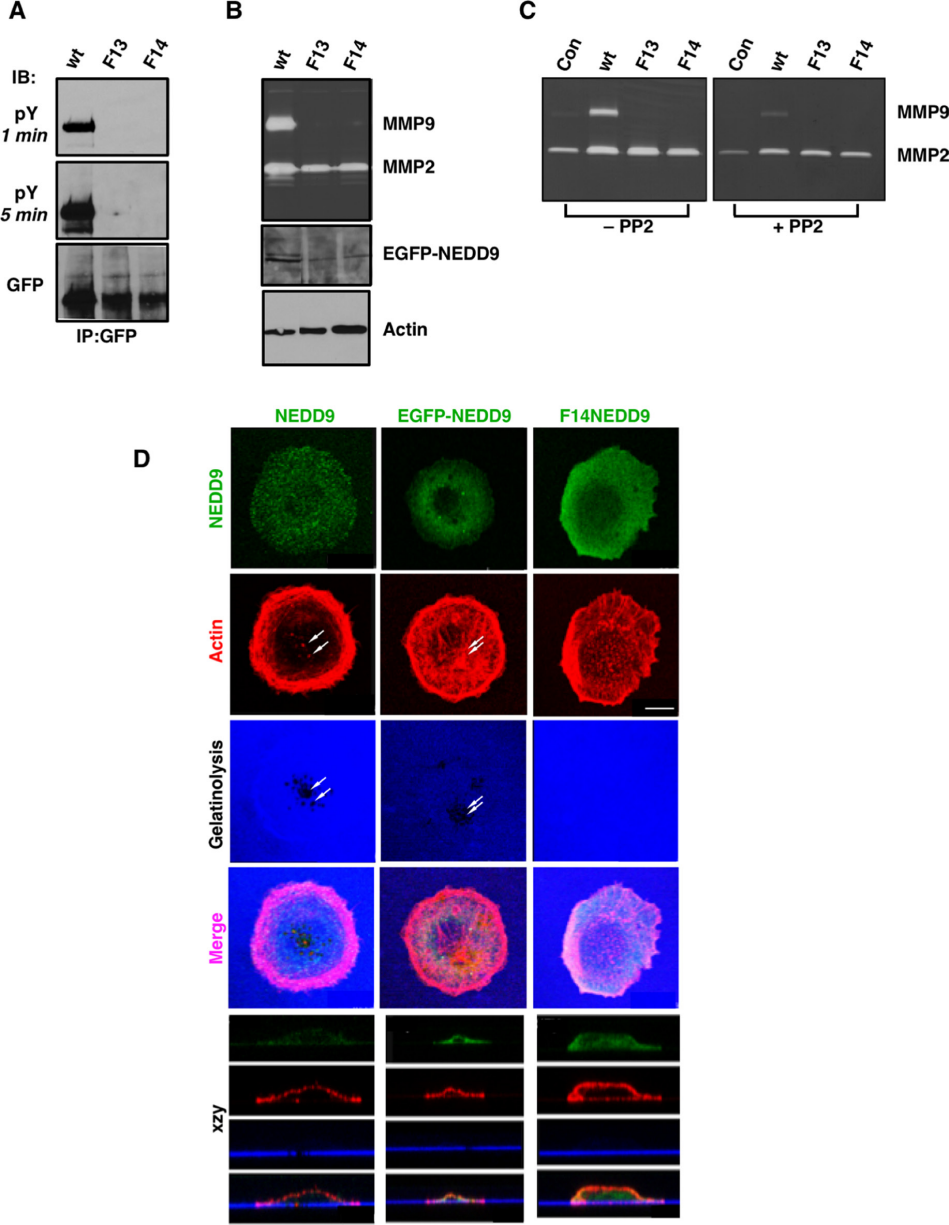
Effect of NEDD9 SD mutations on MMP secretion and invadopodia formation (**A**) F13 and F14 NEDD9 lack the ability to become tyrosine phosphorylated. EGFP-tagged proteins were immunoprecipitated from whole cell lysates, resolved by SDS-PAGE and immunoblotted with anti-pY antibodies or anti-GFP antibodies as indicated. (**B**) In-gel zymography of MMPs (gelatinases) secreted into the medium. Samples were evaluated for transfected proteins by GFP immunoblot and were normalized to cellular actin levels. (**C**) Treatment of cells with the Src family kinase inhibitor PP2 (1 µM) reduces MMP9 secretion and to a lesser extent, MMP2 secretion. (**D**) Invadopodia/*in situ* gelatinase assay of parental SCC9 cells and cells stably expressing WTNEDD9 or F14NEDD9. NEDD9 localization in parental cells was analyzed by immunocytochemistry; WTNEDD9 and F14 NEDD9 were visualized based on GFP fluorescence. Gelatinase activity was determined by proteolysis of Alexa-tagged gelatin-coated coverslips. Evidence of gelatin proteolysis is evident in the xzy stacks for parental cells and cells transfected with WTNEDD9 but not for F14NEDD9 expressing cells. Scale bar; 12 µm.

NEDD9 has a modular domain structure, homologous to that of p130Cas/BCAR1, comprised of an N-terminal SH3 domain (residues 3–65), a substrate domain (SD; residues 110–356), a serine-rich region (SRR; residues 357–560) and a C-terminal focal adhesion (FA) targeting (FAT) domain (residues 561–834 [24]; Figure 1A). NEDD9 contains 29 tyrosine residues, 13 being present within consensus Crk/Nck SH2 domain binding motifs (YxxP) within the SD ([25]; Figure 1B). We previously demonstrated that NEDD9 silencing reduces migration, invasion, MMP secretion and invadopodia formation in HNSCC cells derived from the tongue (SCC9 cells; [11]). To further define the role of the NEDD9 SD in in these processes, all 13 tyrosines were mutated to phenylalanine generating F13NEDD9. We first confirmed that this mutant was incapable of being tyrosine phosphorylated when stably expressed in SCC9 cells (Figure 2A). This lack of tyrosine phosphorylation was also observed for F14NEDD9, which includes the mutation Y629F. F13NEDD9 was also incapable of being tyrosine phosphorylated, whereas Y629FNEDD9 was tyrosine phosphorylated, underscoring that Y629 phosphorylation is not required for SD phosphorylation (Supplementary Figure 1). To further determine the impact of NEDD9 mutation on cell function, we used MMP secretion as a readout. Cells expressing F13NEDD9 or F14NEDD9 exhibited reduced MMP9 secretion compared to WTNEDD9 transfected cells (Figure 2B). In contrast, MMP2 secretion was much less impacted. Treatment of cells with the Src family tyrosine kinase inhibitor PP2 reduced MMP9 secretion with less of an effect on MMP2 secretion (Figure 2C). These results extend our previous findings demonstrating NEDD9 is required for MMP9 secretion, with SD tyrosine phosphorylation being a requirement.

Invadopodia are specialized invadosomes [26] responsible for cancer cell invasion and metastasis. To determine the role of NEDD9 SD tyrosine phosphorylation on invadopodia formation, SCC9 cells stably expressing WTNEDD9 or F14NEDD9 were compared to parental SCC9 cells in invadopodia assays. Invadopodia were identified based on the formation of actin puncta over regions of gelatin digestion, represented as black holes within the substrate underlying the cells (Figure 2D). In control and WTNEDD9 expressing cells, invadopodia formation was observed (Figure 2D), whereas SCC9 cells expressing F14NEDD9 exhibited large numbers of small actin puncta that lacked detectable gelatinolytic activity (Figure 2D). These puncta likely represent precursor invadopodia, which contain invadopodia proteins but lack degradative activity [27]. MMP9 secretion by F14NEDD9 expressing cells was not detectable in keeping with the absence of detectable gelatinolysis (Figure 2B–2D). F14NEDD9 expressing cells were more spread out and polarized front-to-back with apparent lamellipodia and multiple non-productive actin puncta. This morphology contrasted with that of parental and WTNEDD9-expressing cells which were round and exhibited prominent membrane ruffling.

The lack of invadopodia formation in F14NEDD9 expressing cells underscores a key role for NEDD9 in invasive and metastatic signaling paradigms, as well as its requirement for Src-dependent tyrosine phosphorylation. In analogous studies on p130Cas/BCAR1 in breast cancer cells [28], mutation of all 15 of its SD YxxP tyrosines to phenylalanine [29] resulted in reduced proliferation, migration and invasion [30].

### Disruption of the NEDD9 SH3 domain blocks MMP9 secretion and invadopodia formation

**Figure 3:**
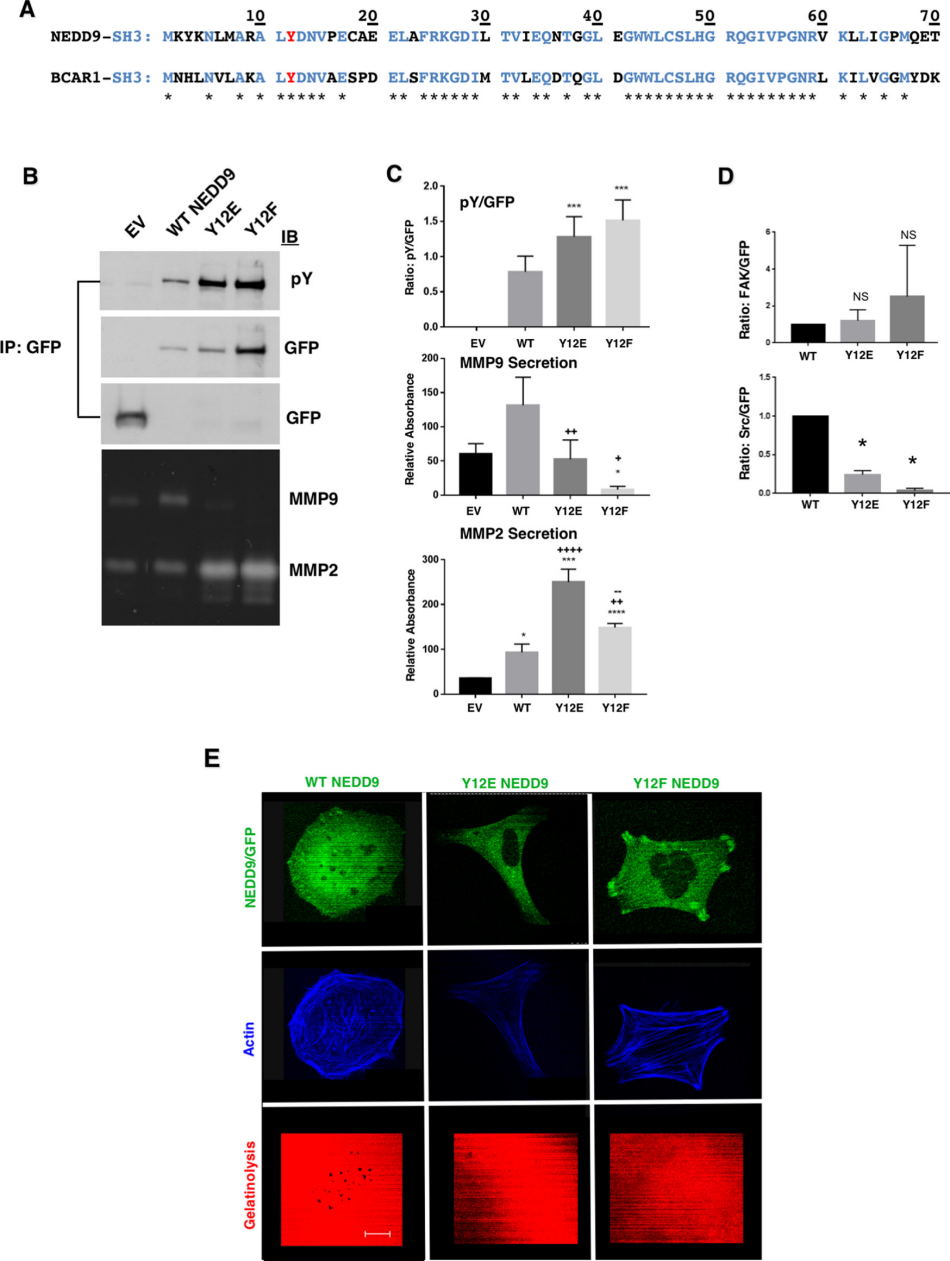
Effect of Y12 mutation on MMP secretion and invadopodia formation (**A**) Sequence comparison of NEDD9 and p130Cas SH3 domains. Y12 is shown in red with residues common to both proteins indicated in blue and with stars. (**B**) Immunoprecipitation (IP) of GFP followed by immunoblot with anti-pY or anti-GFP. The third (GFP) blot represents smaller mol. WT. proteins to enable detection of GFP (30 kDa). (**C**) The immunoprecipitation and zymography experiments shown in B) were performed three times, quantified and analyzed using the student’s *t*-test. For pY/GFP - WT vs Y12E ^***^p < 0.001; WT vs Y12F ^***^p < 0.0001; for MMP9 - EV vs Y12F ^*^p < 0.016; WT vs Y12E ^++^*p* < 0.003; WT vs Y12F ^+^*p* < 0.02; for MMP2: EV vs WT ^*^p < 0.025; EV vs Y12E ^***^p < 0.001; EV vs Y12F ^****^p < 0.0001; WT vs Y12E ^++++^*p* < 0.0001; WT vs Y12F ^++^*p* < 0.007; Y12E vs Y12F ^––^*p* < 0.005. (**D**) The upper graph shows a trend toward FAK preferentially binding to Y12FNEDD9 over Y12ENEDD9 and WTNEDD9 that lacked statistical significance. The association of c-Src with WTNEDD9 over Y12E- and Y12FNEDD9 ^*^p = 0.02. (**E**) Invadopodia assays on cells stably expressing WT, Y12E- and Y12FNEDD9. Invadopodia were only detected in WTNEDD9 expressing cells. Scale bar; 12 µm.

Another feature shared by NEDD9 and p130Cas is an N-terminal SH3 domain, both containing a tyrosine residue at position 12 (Y12; Figure 3A). They are 68.1% identical within residues 1–69 and have the same tyrosine residues Y12 ALYDN(F/V) found in other phosphorylated SH3 domains [31]. In Src-transformed fibroblasts and invasive cancer cell lines p130Cas may be phosphorylated at position Y12 [21]. In the case of NEDD9, anti-phosphotyrosine blotting of F13 and F14NEDD9 revealed the lack of detectable phosphorylation of Y12 or of any other tyrosine (Figure 2A). We generated Y12E and Y12FNEDD9 mutant expressing cells as reported for p130Cas [21] to determine whether these modifications impact MMP9 secretion or invadopodia formation. Both Y12ENEDD9 and Y12FNEDD9 exhibited increased tyrosine phosphorylation compared to WTNEDD9 based on GFP immunoprecipitation and anti-pY immunoblotting (Figure 3B and 3C**)**. The elevated phosphorylation of Y12FNEDD9 is similar to what was observed with Y12Fp130Cas (Figure 3B). These results suggest that substituting glutamic acid or phenylalanine for tyrosine at position 12 in the NEDD9 SH3 domain enhances tyrosine phosphorylation of the SD [31]. MMP9 secretion was significantly reduced in cells expressing either Y12 substitution compared to WTNEDD9 cells; conversely, MMP2 secretion was significantly increased over WTNEDD9 levels in Y12 mutant expressing cells (Figure 3C). Cells expressing these mutants lacked gelatinolytic activity and invadopodia formation consistent with the observed decrease in MMP9 secretion (Figure 3E). In addition to altered signaling to MMP2/9 secretion, NEDD9 Y12 mutant expressing cells exhibited altered morphologic phenotypes. Y12ENEDD9 cells were flat and extended with a mesenchymal morphology (Figure 3E). Y12FNEDD9 expressing cells were more spread out and exhibited significant actin cabling and FAs to which Y12FNEDD9 was localized (Figure 3E). This cell morphology is similar to what was reported for Y12Fp130Cas expressing cells in which FAK exhibited preferential association with this mutant [21]. Based on co-immunoprecipitation analysis the FAK binding activity of Y12ENEDD9 and Y12FNEDD9 cells was equivalent to WTNEDD9 cells, with Y12FNEDD9 trending toward higher FAK association (Figure 3D). However, co-immunoprecipitation analysis revealed that c-Src association with either Y12ENEDD9 or Y12FNEDD9 was significantly less than its association with WTNEDD9 (Figure 3D), underscoring the importance of Src in invadopodia formation [32].

### The SSNEDD9 mutant provides the minimum structural requirements for MMP9 secretion and invadopodia formation

**Figure 4:**
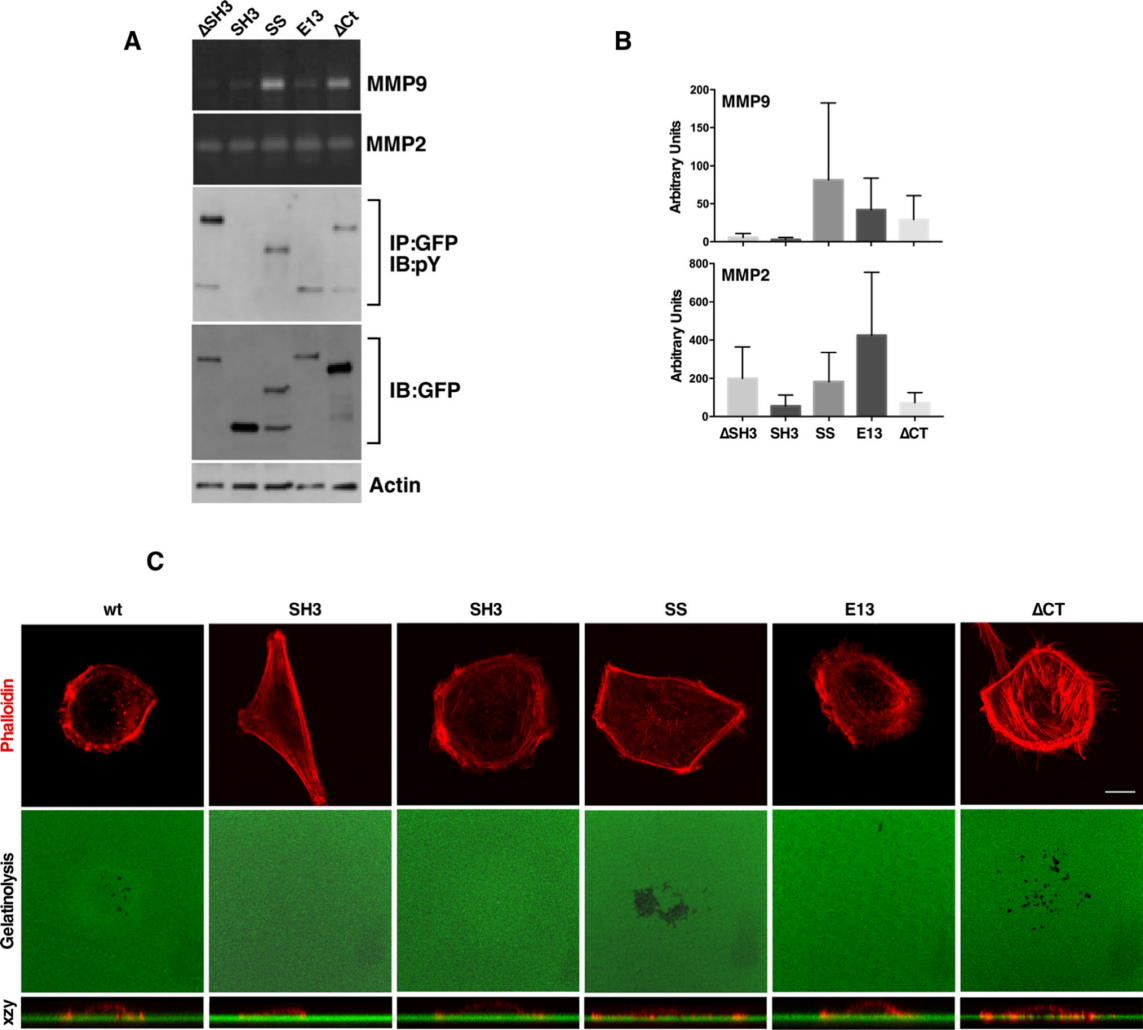
SD tyrosine phosphorylation and an intact SH3 domain are required for MMP9 secretion and invadopodia formation (**A**) ∆SH3, SH3, SS, E13 and ∆CT NEDD9 constructs were evaluated for MMP secretion quantified in three separate experiments in (**B**). (**C**) Confocal images of invadopodia assays confirmed that besides WTNEDD9, SS- and ∆CTNEDD9 enabled invadopodia formation downstream of MMP9 secretion. Scale bar; 7 µm.

We next tested the effect of SH3 domain, C-terminal FAT domain and Serine-rich Region deletions on MMP secretion and invadopodia formation. Deletion of the SH3 domain resulted in cells with a flat, spread-out morphology that lacked MMP9 secretion (Figure 4A and 4B) and invadopodia (Figure 4C), similar to what was observed for the Y12 mutants. Expression of the NEDD9 SH3 domain alone resulted in elevated membrane ruffling and a lack of gelatinolysis and invadopodia formation (Figure 4C). Of note, cells expressing the SH3-SD fragment of NEDD9 (SS/SSNEDD9), lacking both the serine-rich region and C-terminal FAT domain, exhibited strong SD phosphorylation, MMP9 secretion and invadopodia formation (Figure 4A). Additional zymogram analysis of MMP2/9 secretion is provided in Supplementary Figure 2. A summary of the results obtained with all the mutants is shown in Table 1. These findings suggest that an intact SH3 domain and SD tyrosine phosphorylation are the minimal requirements for NEDD9 signaling to MMP9 secretion and invadopodia formation. Reduced MMP9 secretion was strongly associated with a lack of invadopodia formation and was observed in cells with reduced NEDD9 tyrosine phosphorylation and/or a disrupted SH3 domain (Table 1). The preferential association of SH3 mutants with FAK over Src may also contribute to decreased invasive behavior.

**Table 1:** NEDD9 mutant characteristics

NEDD9	WT	F14	E13	Y12E	Y12F	∆CT	SS	SH3	∆SH3
**SD phosphorylation**	+	–	–	+	+	+	+	–	+
**SH3 alteration**	–	–	–	+	+	–	–	–	+
**MMP9 secretion**	+	–	–	–	–	+	+	–	–
**MMP2 secretion**	+	+	+	+	+	+	+	+	+
**Invadopodia**	+	–	–	–	–	+	+	–	–

Impact of the NEDD9 mutations on substrate domain (SD) tyrosine phosphorylation, SH3 domain alteration, MMP2 and MMP9 secretion and invadopodia formation.

### A role for MICAL1 in regulating MMP9 secretion

**Figure 5:**
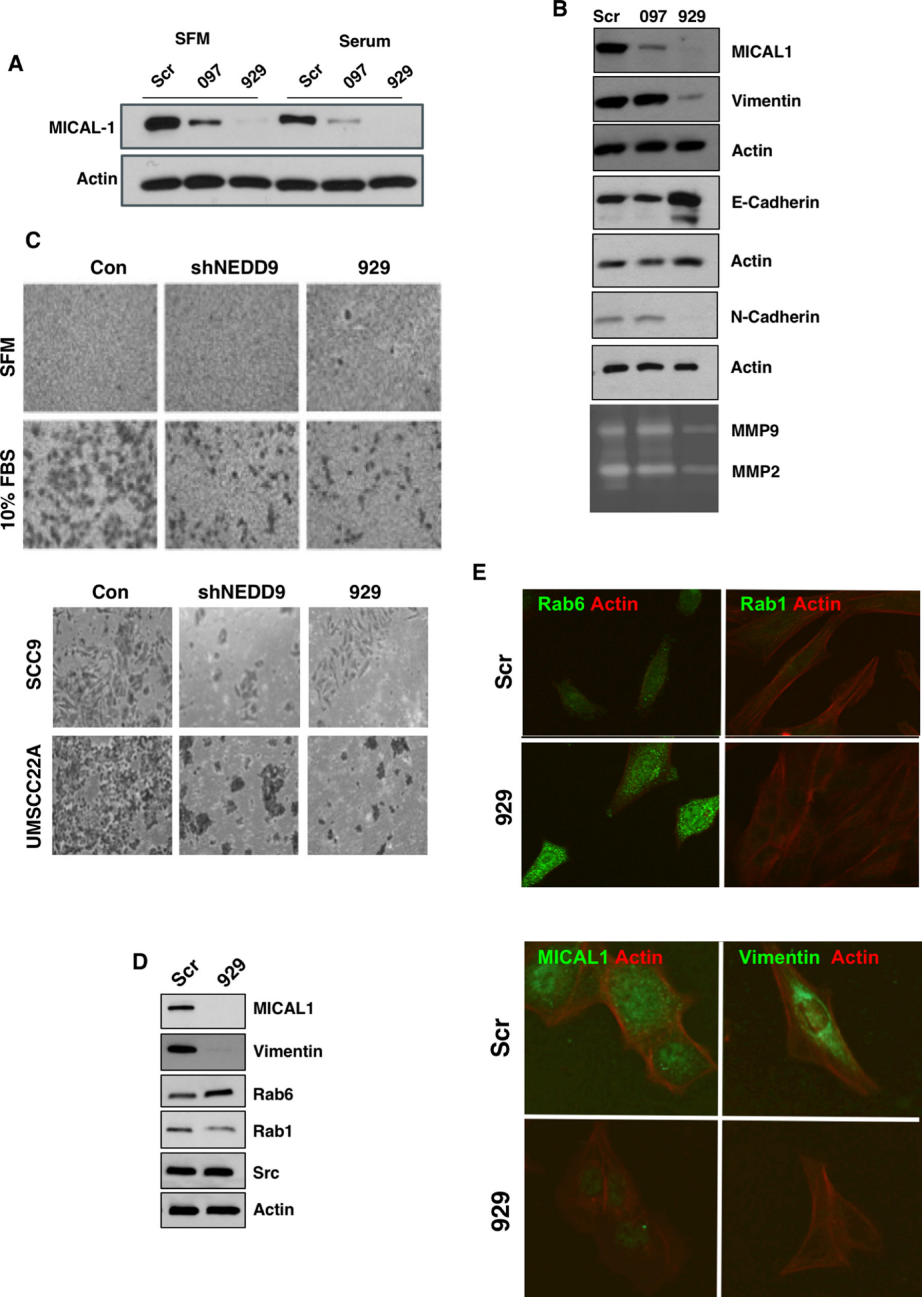
MICAL1 knockdown reduces cell invasiveness and reduced MMP9 secretion (**A**) SCC9 **c**ells were stably infected with two different MICAL1 shRNA species, 097 and 929. (**B**) MICAL1 silencing reduced vimentin, N-cadherin and MMP2/9 secretion, while elevating E-cadherin levels. (**C**) MICAL1 silencing reduced invasion to the same extent as NEDD9 knockdown in SCC9 cells (*upper and lower*) and UMSCC22A cells (*lower*) [11]. (**D**) MICAL1 silencing increased Rab6 and decreased Rab1 levels. (**E**) Immunocytochemical verification of elevated Rab6 and reduced vimentin expression levels in MICAL1-silenced cells.

**Figure 6:**
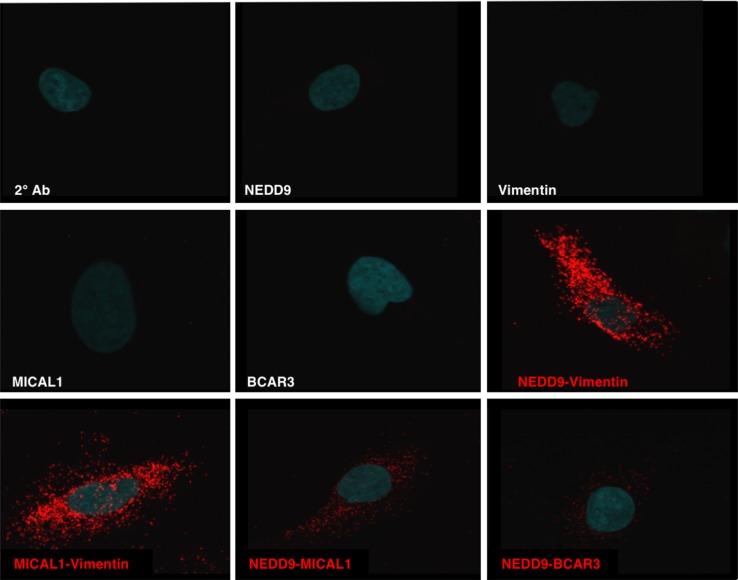
NEDD9 interactions defined by Proximity Ligation Assay Specific interactions (red) of NEDD9 with MICAL1, BCAR3 and vimentin along with the specific binding of MICAL1 and vimentin as determined by proximity ligation assay (PLA).

Based on the requirement of an intact SH3 domain for NEDD9 mediated MMP9 secretion and invadopodia formation we tested whether MICAL1, molecule interacting with Cas L1, plays a role in MMP secretion and invadopodia formation. MICAL1 is a monooxygenase-containing protein reported to mediate growth cone collapse and axonal repulsion via redox signaling to effect axon guidance [33] originally discovered as a NEDD9 SH3 domain interacting protein [34]. We therefore silenced MICAL1 in SCC9 cells using small hairpin RNA (shRNA). As shown in Figure 5A, two different MICAL1 targeting shRNAs silenced MICAL1 expression with the 929-construct completely eliminating MICAL1 protein expression and the 097-construct caused ∼90% loss of MICAL1. Cells silenced with the 929-MICAL1 shRNA exhibited reduced MMP9 and MMP2 secretion, reduced vimentin and N-cadherin levels and increased E-cadherin levels (Figure 5B). This change in MMPs and protein expression is consistent with the reduced invasion capability of these cells and a switch from an invasive, mesenchymal to an epithelial phenotype (Figure 5C). In more recent studies Rab6-decorated vesicles trafficking between the Golgi and plasma membrane were found to accumulate within the growth cones of MICAL1 knockout mice [35]. As shown in Figure 5D and 5E, MICAL1 silenced cells exhibited elevated Rab6 levels, as reported for MICAL1 knockout mice (Figure 5D and Supplementary Figure 3). Using proximity ligation assay (PLA) analysis we observed a specific interaction between NEDD9 and MICAL1 (Figure 6). However, in GFP co-immunoprecipitation experiments were unable to demonstrate a specific interaction between the NEDD9 SH3 domain and MICAL1 (Supplementary Figure 4) as initially described by Suzuki *et al.*, [34]. We were able to demonstrate a MICAL1-vimentin interaction as described by Suzuki *et al.*, [34] by proximity ligation analysis. Taken together, these results suggest that MICAL1 and NEDD9 may interact physically and/or functionally, with both proteins contributing to the invasive phenotype by stimulating vesicle trafficking, MMP9 secretion and invadopodia formation.

## DISCUSSION

To define the role of NEDD9’s functional domains in HNSCC cell invasion we used MMP9 secretion and invadopodia formation as functional readouts of invasive cell behavior. Replacement of the 13 YxxP motif tyrosines in the SD along with Y629 to phenylalanines reduced MMP9 secretion with a negligible effect on MMP2. This alteration also limited the ability of cells to form invadopodia, suggesting that MMP9 secretion may be required for invadopodia maturation and that MMP2 is not. The essential requirement for a functional SH3 domain in MMP9 secretion and invadopodia formation was also confirmed by its deletion. ∆SH3NEDD9 lacked activity in both assays. Collectively, the results obtained indicate that SD tyrosine phosphorylation in conjunction with a functionally intact SH3 domain are both necessary for MMP9 secretion and invadopodia formation. MMP2 secretion was relatively unaffected by the NEDD9 modifications evaluated, reflecting the differential regulation of these MMPs [36].

Invadopodia formation was dependent upon MMP9 secretion and was not supported by MMP2 secretion. ∆CTNEDD9 and SSNEDD9 expression each resulted in SD phosphorylation, MMP9 secretion and invadopodia formation, indicating that the C-terminal domain and serine-rich region are dispensable with respect to these activities. Relative to these findings, mutation of Y189 to phenylalanine, (equivalent to Y253 of p130Cas), was reported to enhance cell migration with substitution to Y189D being inhibitory [37]. This differs somewhat with our findings for MMP9 secretion and invadopodia formation but is consistent with the inability of E13NEDD9 to support these activities.

Substituting glutamic acid or phenylalanine at position Y12 within the SH3 domain was based on a report where these substitutions in p130Cas decreased or increased tyrosine phosphorylation, respectively, and in which Y12Fp130Cas expression caused increased FA formation [21]. Increased FA formation was attributed to increased association of Y12Fp130Cas with FAK. We did not observe a significant increase in FAK association with either Y12 NEDD9 mutant. Instead, both mutants exhibited increased tyrosine phosphorylation compared to WTNEDD9. On the other hand, MMP9 secretion was significantly reduced and MMP2 secretion was enhanced by Y12 substitution. Neither Y12 mutant supported invadopodia formation consistent with reduced in MMP9 release. We previously reported that NEDD9 is preferentially activated by Src over FAK [11], reflecting a role for Src family kinases in invadopodia formation [38]. Related to this, Ahn *et al.*, showed that Src is required for NEDD9 signaling to mesenchymal over amoeboid movement [39] and Bradbury and coworkers [12] demonstrated an exchange of Src and FAK at FAs modulated by NEDD9. Finally, Wang and McNiven [19] reported that FAs exhibit degradative activity that is activated by p130Cas recruitment of FAK to these sites at the plasma membrane. Taken together, the present findings support a role for NEDD9 activity in invasive behavior.

Y12FNEDD9 and Y12ENEDD9 each exhibited enhanced SD tyrosine phosphorylation, decreased MMP9 and increased MMP2 secretion. These findings suggest that SD tyrosine phosphorylation is not sufficient for MMP9 secretion and invadopodia formation. The lack of MMP9 secretion observed may be due to Y12FNEDD9 being preferentially localized to FAs. Alternatively, SH3 domain and SD interactions may both be required for MMP9 secretion and invadopodia formation. This may reflect Src binding to the SH3 domain in WTNEDD9 expressing cells. These findings support the requirement for MMP9 secretion in order for cells to form invadopodia. Our results are in accord with the known differential regulation of MMP2/9 [36, 40–43] and their discrete targeting/localization [43, 44], with MMP9 secretion being regulated [45] and MMP2 having a more constitutive release profile [46–49]. Using breast cancer cell lines, McLaughlin and coworkers [50] reported that NEDD9 depletion reduced MMP2 and MMP9, cell invasion and matrix degradation. Moreover, they found that depleting NEDD9 reduces MMP14 levels via TIMP2 inactivation. While we did not examine MMP14 expression in this study, we previously reported that MMP14 localizes to invadopodia membranes and that NEDD9 silencing reduces MMP14 and MMP2/9 expression, cell invasion and invadopodia formation in HNSCC cells [11].

Based on the notion that invadopodia are sites of focal MMP9 secretion, we examined a role for MICAL1 involvement downstream of NEDD9. MICAL1 was originally isolated as a NEDD9 binding partner [34]. The MICAL family of flavoprotein monooxygenase domain containing redox proteins consists of three species (MICAL 1–3) that regulate actin dynamics and growth cone collapse in *Drosophila* [33], the distribution of secretory vesicles in neurons [35] and Rab binding to secretory vesicles [51]. MICAL1 silencing in SCC9 cells resulted in elevated Rab6, reduced Rab1 and a concomitant loss of vimentin, similar to what was seen in cultured MICAL1 null dorsal root ganglion cells where Rab6 accumulates within cells and IgCAM trafficking to the cell surface was reduced [35]. Reduced Rab1 levels and a commensurate decrease in MMP9 secretion by cultured human oral squamous cell carcinoma cells has been reported [52]. The decrease in vimentin levels observed in MICAL1 silenced cells may contribute to reduced invasive cell programming including reduced MMP9 secretion consistent with MET. In turn, Rab1 levels may be reduced to conserve ER/Golgi membranes or to reduce autophagy [53].

## CONCLUSIONS

Elevated expression of MMP2 and MMP9 is associated with higher rates of local recurrence and regional/distant metastasis in HNSCC of the tongue with MMP9 having the highest specificity [54]. MMP9 is similarly associated with the metastasis of Lewis lung carcinoma cells in mice downstream of VEGFR-1 activation [55]. The present studies support a role for NEDD9 as an enhancer of MMP9 secretion, invadopodia formation and cancer cell invasion. While the role of MICAL1 is less clear, our findings in HNSCC cells and those of Deng and coworkers in breast cancer [56] suggest that reduced MICAL1 supports mesenchymal to epithelial transition. This is consistent with our prior demonstration that overexpression of NEDD9 leads to epithelial to mesenchymal transition [11]. Future studies will be required to identify these inter-relationships.

## MATERIALS AND METHODS

### Reagents and plasmids

DMEM and mitomycin C were from Sigma (St. Louis, MO). BCA reagent was obtained from Pierce (Rockford, IL). The NEDD9 construct was generously provided by Dr. Erica Golemis (Fox Chase Cancer Center). All NEDD9 plasmids contained an N-terminal EGFP. F13, F14, Y12E, Y12F, SS, SH3and ∆SH3 were generated using WTNEDD9 and confirmed by DNA sequencing; ∆CTNEDD9 and BCAR3 were from Dr. K. O’Neill, Australia. All other chemicals were of reagent grade or higher.

### Antibodies

Monoclonal 4G10 Anti-phosphotyrosine (pY) (1:1000 for WB) and anti-actin C4 (1:10,000 for WB) antibodies were obtained from Millipore (Temecula, CA). Polyclonal anti-Rab1 FL205 (1:500 for WB and 1:250 for IF), anti-Rab6 C19 (1:500 for WB and 1:250 for IF), anti-E-Cadherin H108 (1:500 for WB) antibodies and monoclonal NEDD9 (2G9) (1:250 for PLA and IF and 1:250 for IP) and anti-vimentin RV202 (1:500 for WB, 1:250 for IF and PLA) antibodies were from Santa Cruz Biotech (Santa Cruz, CA). Polyclonal anti-MICAL1 antibody (1:1000 for WB, 1:100 for IF and PLA) was obtained from Proteintech (Rosemont, IL). Polyclonal anti-NEDD9 C-terminal (1:500 for IF and PLA) antibody was from Sigma-Aldrich (St-Louis, MO). Polyclonal N-Cadherin D4R1Hxp (1:1000 for WB) antibody and monoclonal anti-Scr L4A1 (1:1000 for WB) were obtained from Cell Signaling Technology (Danvers, MA). Polyclonal anti-BCAR3 (1:2000 for WB and 1:200 for PLA) was from Bethyl Laboratories (Montgomery, TX). Polyclonal anti-GFP (1:5000 for WB) was generously provided by Dr. Scott T. Eblen (Medical University of South Carolina, Charleston, SC). Monoclonal anti-GAPDH 6C5 (1:10,000 for WB) was obtained from Abcam (Cambridge, MA). Polyclonal anti-GFP (1:500 for IP) was from Genetex (Irvine, CA). HRP-conjugated goat-anti-mouse (1:5000 for WB) and goat-anti-rabbit (1:1000 for WB) secondary antibodies were obtained from Jackson ImmunoResearch Laboratories (West Grove, PA). HRP-conjugated donkey-anti-chicken (1:7500 for WB) secondary antibody was obtained from Thermo Fisher Scientific (Waltham, MA).

### shRNA and cell culture

SCC9 and SCC25 cells (ATCC, Manassas, VA; UMSCC22A and UMSCC22B cells (Dr. Thomas Carey, University of Michigan) were maintained as described elsewhere [11, 57]. Cell lines stably expressing mutants were selected with Geneticin^®^ and sorted. Mission^®^ shRNAs in the pLKO.1-Puro were packaged into lentivral particles in HEK293T cells followed by transduction into cell lines as described by the manufacturer. For plasmid DNA, cell suspensions were transfected with Nanojuice^®^ (EMD Millipore) according to the manufacturer’s protocol.

### Immunoblot analysis

Standard immunoblot analysis of whole cell lysates was carried out as previously described [58].

### Invasion assay

Serum-starved cells were plated in the top chamber of matrigel-coated transwell filters in 24 well plates at a density of 30 × 10^3^ cells/well for 1 h. SFM in the upper and lower chambers was exchanged for fresh SFM containing the treatment indicated and cells incubated for 24 h. Each filter was rinsed with PBS and stained with a 1:10,000 dilution of Hoechst dye in PBS for 10 min. The top portion of each filter was swabbed with a Q-tip and washed three times with PBS. Cells on the filter bottoms were imaged with a DAPI filter and nuclei were counted using Cell Profiler v1.0.4942 [59].

### Gelatin zymography

Secreted MMPs in conditioned medium were affinity adsorbed with gelatin-Sepharose as previously detailed [11, 60]. SDS sample buffer lacking DTT was added and proteins were resolved on 10% acrylamide, SDS gels containing polymerized gelatin (0.5–2 mg/ml). MMPs were renatured using two 30 min detergent exchange washes. Gels were incubated for 6–48 h at 23°C to allow MMP to degrade the gelatin followed by staining with Coomassie brilliant blue. Destained gels were scanned and inverse images were quantified using ImageJ, with relative absorbance values for MMP9 or MMP2 normalized to cell lysate GFP/actin ratios and EV or WT value set to 1.0.

### Invadopodia assay

Coverslips (#1) were coated with 80 µl of warmed gelatin (comprised of fluorescently labeled:unlabeled gelatin or fibronectin at a 1:8 ratio) as previously described [11]. Coated coverslips were allowed to solidify and dry to a thin film at 23°C in the dark and cross-linked with 4% paraformaldehyde for 10 min, reduced with 1 ml of 10% Na borohydride, and washed 3 times with PBS. Coverslips were then equilibrated in SFM containing 10 µM GM6001 for at least 15 min. SCC9 cells were trypsinized with 0.25% trypsin-EDTA, suspended and mixed with sterile 2.5% bovine serum albumin (BSA) to neutralize trypsin, and centrifuged at 500 x g for 5 min. Cells were resuspended in SFM and coverslips seeded at a low density (final GM6001, 5 µM). Cells were allowed to adhere overnight and attached cells were gently washed with SFM. Invadopodia were defined as foci of gelatin degradation/regions of decreased gelatin fluorescence and increased F-actin fluorescence intensity.

### Immunocytochemistry

Cells seeded on coverslips were fixed in 4% paraformaldehyde, permeabilized with 0.1% Triton X-100 and blocked with 1% BSA in PBS for at least 30 min. 100 µl of primary antibody diluted in 1% BSA and 0.1% Triton X-100 in PBS was added for 1 h followed by washing and secondary antibody (1:200) addition for 1 h. Coverslips were rinsed with PBS, inverted onto a glass slide with anti-bleaching sealant and sealed with fingernail polish. Labeled cells were imaged on an Olympus FluoView FV10i laser scanning confocal microscope with 405, 473, 559 and 635-nm lasers, in the xyz and xzy planes or with a Leica TCS SP2 AOBS laser scanning confocal microscope with 458, 477, 488, 514, 543 and 633-nm lasers. Images were prepared for publication using Olympus FluoView, Leica Imaging and ImageJ Fiji software.

### Statistical analysis

Summary statistics for all continuous variables are presented as means and standard deviations. Categorical data are summarized as frequencies and percentages. Differences in baseline characteristics between the local disease group and advanced disease group were analyzed using the Student’s *T*-test.

## SUPPLEMENTARY MATERIALS FIGURES



## Abbreviations

BCAR1: breast cancer anti-estrogen resistance 1
BCAR3: breast cancer anti-estrogen resistance 3
CasL: Crk-associated substrate in Lymphocytes
ECM: extracellular matrix
EGFP: enhanced green fluorescent protein
FA: focal adhesion
FAK: focal adhesion kinase
FAT: focal adhesion targeting
GFP: green fluorescent protein
HEF1: human enhancer of filamentation
HNSCC: head and neck squamous cell carcinoma
HRP: horseradish peroxidase
IF: Immunofluorescence
IP: Immunoprecipitation
MICAL1: molecule interacting with Cas-L1
MMP: matrix metalloproteinase
MT1-MMP: membrane type 1-MMP
NEDD9: Neural precursor cell expressed developmentally downregulated 9
PLA: proximity ligation assay
pY: phosphotyrosine
Rab: Ras-related proteins in brain
SD: substrate domain
shRNA: short hairpin RNA
SRR: serine-rich region
VEGFR: vascular endothelial growth factor receptor
∆CT: deleted C-terminal domain
∆SH3: deleted SH3 domain
