# Supplementary material for: NEDD9 stimulated MMP9 secretion is required for invadopodia formation in oral squamous cell carcinoma
Source: Oncotarget. 2018 May 22;9(39):25503–16. doi: 10.18632/oncotarget.25347 (PMC5986644; doi:10.18632/oncotarget.25347)
Supplement: Supplementary file 1 [file oncotarget-09-25503-s001.pdf]

NEDD9 stimulated MMP9 secretion is required for invadopodia formation in oral squamous cell carcinoma

SUPPLEMENTARY MATERIALS

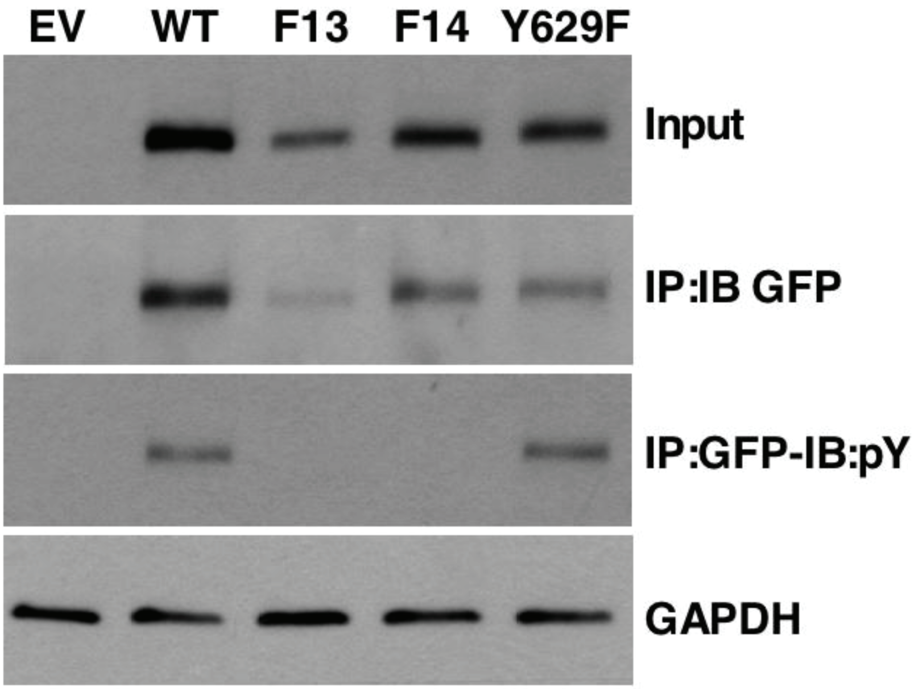

Supplementary Figure 1: Y629F mutation of NEDD9 does not block SD phosphorylation. These results suggest that mutation of Y629F and blocking its ability to be phosphorylated does not impede phosphorylation of the SD, indicating it is not required for SD phosphorylation. Note: in the EV lane, GFP (in SFM ~30 kDa) migrates further below region displayed.

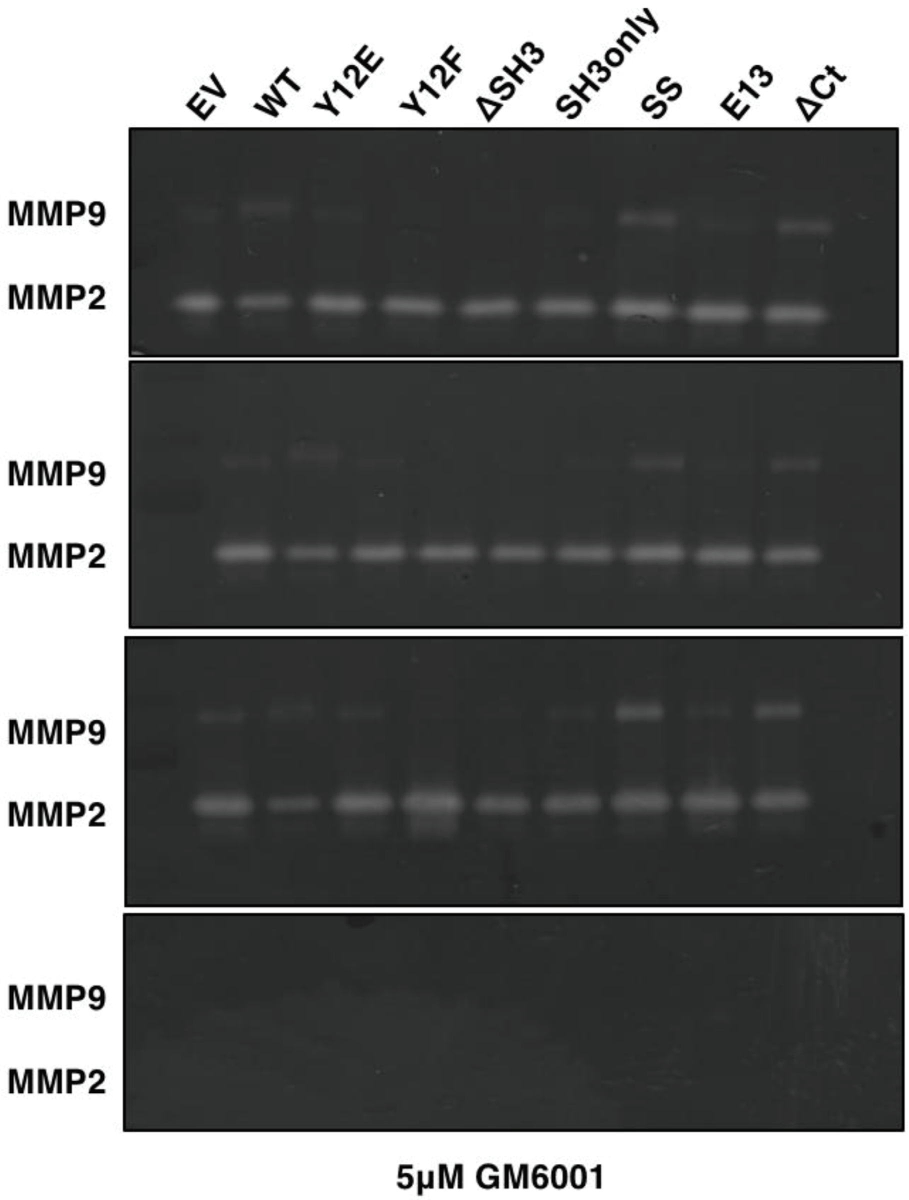

Supplementary Figure 2: NEDD9 mutation impacts MMP9 secretion. Shown are additional zymography assays. Also shown is that addition of the broad-spectrum MMP inhibitor GM6001 during incubation of the gel blocks MMP activity resulting in the loss of detectable *in situ* gelatinolysis activity.

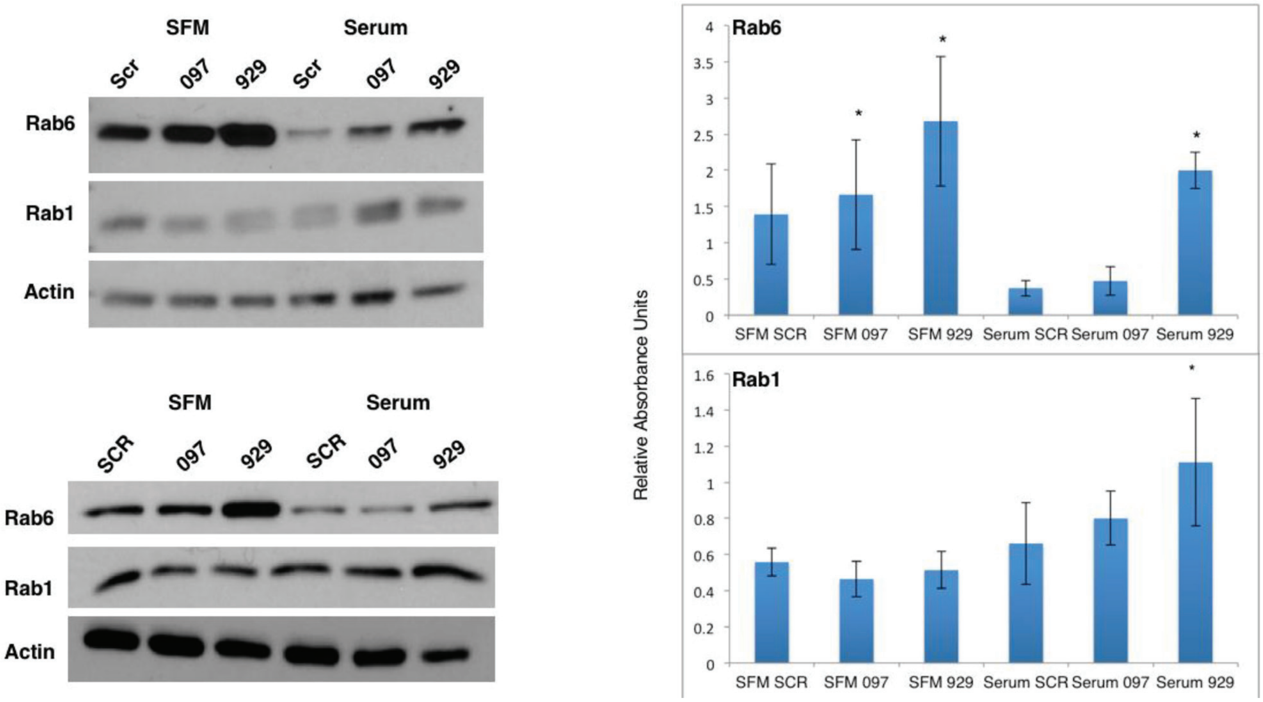

Supplementary Figure 3: Mical1 silencing results in elevated Rab6 levels. Shown (*Left*) are immunoblots of Rab6 and Rab1 and their quantification (*Right*). Cells grown in serum-free medium (SFM) exhibited higher overall levels of Rab6 consistent with reduced trafficking and secretion of MMP9. Rab1 levels were lower in Mical1 silenced cells grown in SFM; Rab1 was elevated in Mical1 null cells grown in serum; $^*p < 0.05$.

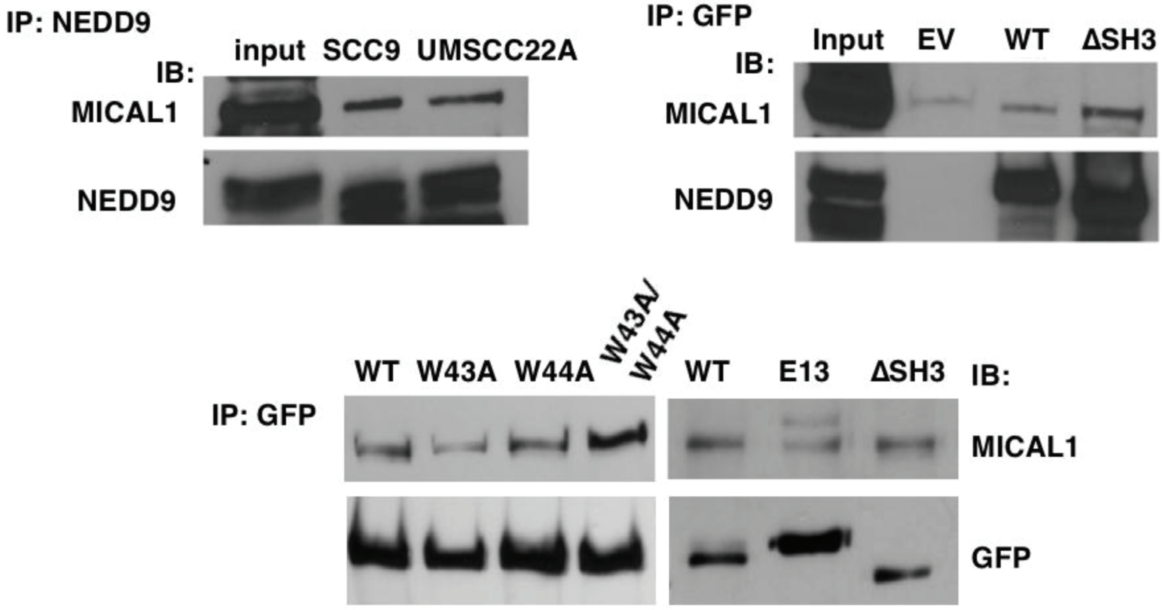

Supplementary Figure 4: NEDD9:Mical1 co-immunoprecipitation persists in the presence SH3 domain mutations or deletion. Suzuki *et al*., [35] identified Mical1 as a NEDD9 binding partner by Far Western blotting utilizing a radioactively labeled NEDD9 SH3 domain. It contains a C-terminal PKPP that that targets SH3 domains. Neither mutating the WW domain within the SH3 domain within or deletion of the SH3 domain eliminated the Mcal1-NEDD9 interaction. We were unable to demonstrate that a specific SH3 domain interaction.
